# Case Report: Dislocation Into Vitreous Cavity and Removal of a Posterior Chamber Phakic Intraocular Lens

**DOI:** 10.3389/fmed.2021.792253

**Published:** 2022-01-28

**Authors:** Jingliang He, Li Zhang, Fang Zheng, Xiaoyun Fang

**Affiliations:** Eye Center, The Second Affiliated Hospital, Zhejiang University School of Medicine, Hangzhou, China

**Keywords:** dislocation, removal, posterior chamber phakic intraocular lens, EPRL, PPV

## Abstract

**Purpose:**

To report a rare case of delayed dislocation of a novel posterior chamber phakic intraocular lens into the vitreous cavity, which was successfully treated by a reformed technique.

**Case Presentation:**

A 29-year-old female received Ejinn phakic refractory lens (EPRL) implantation to correct her high myopia. Spontaneous dislocation into the vitreous cavity occurred 26-months post-operatively without traumatic history. Pars plana vitrectomy combined with cutting the EPRL into two equal pieces was performed to remove the dislocated EPRL.

**Conclusion:**

Dislocation into the vitreous cavity of EPRL can be successfully and easily removed by our reformed technique. Concerns about zonules-related complications pre-operatively, intraoperatively, and post-operatively must be raised in the practice of EPRL implantation.

## Introduction

Photoablative refractive surgery is usually a preferred option for ametropia correction. However, complications, such as keratectasia, limit its use in correcting high refractory error (1–3). Implantation of posterior chamber phakic intraocular lens (PC-pIOL) was developed to solve this problem and has become an effective and safe procedure to correct high ametropia when corneal photoablation is not suitable for the patients (4). Implantable Collamer lens (ICL; STAAR Surgical Co., Monrovia, CA, USA), which can correct myopia between −3D to −18 diopters (D), is the most widely implanted PC-pIOL. However, there is, so far, no model of ICL that is designed to correct myopia over −18D. Recently, a novel PC-pIOL, Ejinn phakic refractory lens (EPRL), which can correct up to −30D myopia, was approved by the Chinese Food and Drug Administration (CFDA) in 2017. This product makes it possible to correct myopia over −18 D with a single surgery. However, the long-term safety and effectiveness of EPRL are still needed to be further verified. Here, we present a case of delayed dislocation of EPRL into the vitreous cavity, which was successfully treated by a reformed technique.

## Case Presentation

A 29-year-old female visited our center for refractive surgery. Her manifest refraction was −20.5 −1.0 × 180° in the right eye and −21.0 −1.5 × 4° in the left eye with corrected distance visual acuity (CDVA) of 20/30 and 20/32, respectively. Axial length was 30.30 mm in the right and 30.04 mm in the left eye. The keratometry was 40.52@175/41.82@85 and 41.11@6/42.19@96, respectively. The white to white was 12.1 mm in both eyes. After excluding the traumatic history and systemic diseases, EPRL (Aijinglun Technology Co., Hangzhou, China) implantation was suggested considering the myopia of the patient was beyond −18D, which is the correction limit of ICL.

Ejinn phakic refractory lens (EPRL) model BK113 of −20.5D (11.3 mm diameter) and −21.0D (11.3 mm diameter) were implanted in both eyes, respectively, under topical anesthesia. Laser peripheral iridectomy was performed 3 months before surgery. No immediate complications occurred during the operation. At 1 month follow-up, the uncorrected distance visual acuity (UDVA) was 20/20 bilaterally. Slit-lamp examination and ultrasound biomicroscopy (UBM) showed the centered and proper location of EPRL. Intraocular pressure was 15 and 18 mmHg in both eyes.

**Figure 1:**
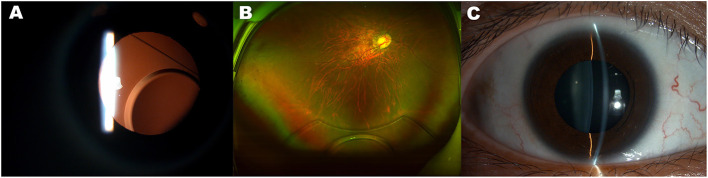
Dislocation of the Ejinn phakic refractory lens (EPRL). **(A)** Slit-lamp examination shows subluxation of the EPRL, which was observed when the patient visited our center due to complaining of suddenly blurred vision of right eye at 26 months post-operatively. **(B)** Luxation into the vitreous cavity of the EPRL revealed by fundus photography. **(C)** Slit-lamp examination shows the position of crystalline lens after the luxation into the vitreous cavity of the EPRL.

**Figure 2:**
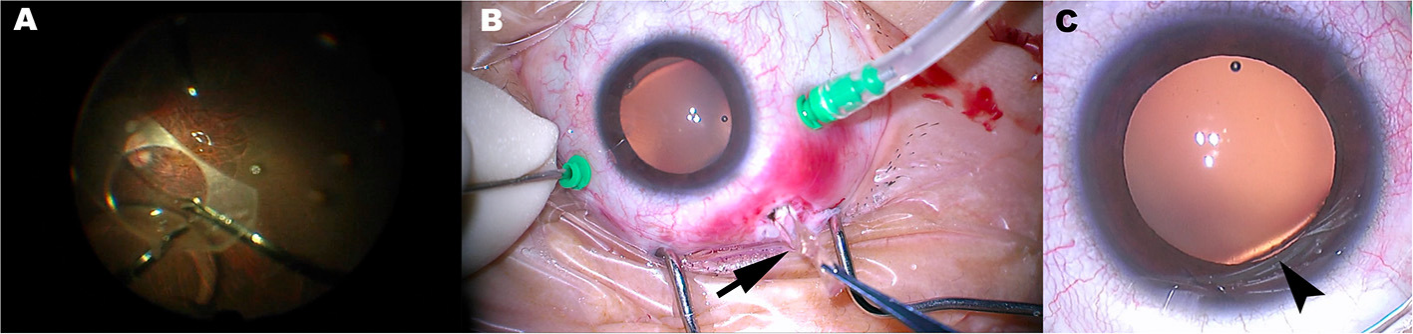
The technique of EPRL removal and the rupture of zonular fibers. **(A)** The EPRL was grasped and cut into two equal pieces along the long axis. **(B)** Half of EPRL (black arrow) was extracted through a 2.5 mm sclerotomy. **(C)** Rupture of zonular fibers and focal dislocation of the lens at nasal inferior (black arrowhead) was observed through an operating microscope when the patient lay on the operating table.

Twenty-six months post-operatively, the patient presented at our center complaining of suddenly blurry right eye vision. The UDVA was 0.15. Slit-lamp examination showed dislocation of the EPRL (Figure 1A). Removal of dislocated EPRL through clear cornea incision was arranged. However, the dislocated EPRL disappeared from the posterior chamber when she came to our center for surgery 2 days later. No phacodonesis or significant zonular weakness was observed in this eye (Figure 1C). Fundus examination showed a luxated EPRL located in front of the retina (Figure 1B). Pars plana vitrectomy, using diffuse illumination through 23-gauge cannulas, was performed to remove the EPRL. After careful vitreous dissection, fluorinated perfluorocarbon (Carl Zeiss Meditec AD, Germany) was injected to make the EPRL detach from the retina. Once floating on the top of the fluorinated perfluorocarbon bubble, the EPRL was grasped and cut into two equal pieces along the long axis (Figure 2A). Then, foreign forceps were used to gently grab each piece and remove them from the vitreous cavity (Figure 2B) through temporal sclerotomy, which was enlarged into 2.5 mm. Sclerotomies were sutured after the examination of the retina (Supplementary Video 1).

At 6-month follow-up, the manifest refraction was −20.0 −1.5 × 176° in the right eye with CDVA of 20/30. The refractive error of this eye was corrected by wearing a contact lens.

## Discussion

Phakic intraocular lens (pIOL) implantation is a refractive surgery for patients who are not suitable for corneal photoablation. It provides high visual quality, preservation of accommodation of patients, and potential reversibility. Several angle-supported pIOLs and iris-fixated pIOLs were developed at the beginning. However, these products quit from the market due to complications such as loss of corneal endothelial cells and uveitis (5, 6). EPRL is one of two commercially available PC-pIOL in China. Comparing with ICL, EPRL has a broader range of myopia correction and is usually implanted in eyes over −18D. In this context, more attention should be paid to zonules-related complications due to the fragile nature of zonular fibers in high myopia eyes. Our report presents a case of EPRL dislocation into the vitreous cavity. To our best knowledge, this is the first report of the luxation of EPRL into the vitreous cavity.

Twenty-six months after being implanted, the EPRL partially dislocated nasal-inferiorly, following its complete disappearance from the posterior chamber 2 days after the initial dislocation was observed. Even though zonular dehiscence or phacodonesis was not observed using slit-lamp examination or UBM before pars plana vitrectomy, focal dislocation of the lens at the inferior nasal quadrant was observed through an operating microscope when the patient laid on the operating bed (Figure 2C). The position of focal lens dislocation is consistent with that of initial partial luxation of EPRL, suggesting that the route of EPRL luxated into the vitreous cavity is through the gap of broken zonular fiber. The reason why we did not observe the focal dislocation of the lens by slit-lamp examination before surgery might be due to the body position. At sitting position, the gravity of the lens counteracted the unbalance strength resulting from the weakening of inferior zonular fibers, causing inferior dislocation of lens unnoticeable.

The exact reason for the focal zonular rapture is unknown, but some speculations may explain this process. Occult defect of zonules prior to the surgery may account for this complication after EPRL implantation. Despite the denial of traumatic history, forgotten slight ocular injury might also cause damage to zonular fiber. The aforementioned zonular deficiencies do not usually result in clinical manifestations. Zonular defects without signs have also been found in cataract surgery. Gimbel et al. (7) reported that 8 of 14 eyes in patients who received insertion of capsular tension ring for zonular weakness or defects during cataract surgery had no signs of zonular loosen or broken before operation. On the other hand, zonular weakness is common in high myopia eyes. It was very likely that zonular loosening or weakness exists pre-operatively in this patient. Intraoperative manipulation, such as overfilling the anterior chamber using viscoelastic agent and rotating the haptics of EPRL, may be easier to damage the zonular fibers of high myopic eyes than that of normal eyes.

The position of the zonular rapture is just opposite to the main incision that the EPRL was implanted through. The zonular was possibly damaged by the haptics of EPRL when it was inserted into the posterior chamber. This iatrogenic trauma to zonular fibers was difficult to observe during the surgery because the iris shielded the zonular fibers. Considering the floating design of EPRL, it was likely to rotate in the direction of the zonular broken quadrant from the original horizontal position (8, 9). In addition, the soft and slipper property of EPRL also increased the possibility of it falling into the vitreous cavity through the broken zonules.

To avoid pupillary block, peripheral laser iridotomy (PLI) at the position of 10:30 and 13:30 was performed before EPRL implantation. Usually, PLI is a safe treatment with a very low possibility of minor complication occurrence. Some cases of lens subluxation related to PLI still have been reported (10, 11), indicating that the PLI may damage zonular fibers around the related site. In this case, the focal dislocation of the lens was located at the inferior nasal quadrant, which is far from the site of PLI. Therefore, it is unlikely that the zonular rupture was caused by PLI.

Exchange of EPRL was not considered due to the risk of reoccurrence of EPRL dislocation. The luxated EPRL was eventually removed by pars plana vitrectomy. Even though a previous case report presented that phakic refractive lens (PRL) dislocated into vitreous cavity was integrally removed through a 3.5 mm sclerotomy (12), in our case, it was difficult to extract the entire EPRL through a small sclerotomy due to the frangibility of this purified silicone made PC-pIOL. Hence, we improved the removal technique of dislocated PC-pIOL from the vitreous cavity in the phakic eye. After vitreous dissection, the EPRL was cut into two equal pieces using intraocular micro-scissors along the long axis. This manipulation made it possible that the EPRL can be removed out through a sclerotomy as small as 2.5 mm and reduce the damage upon uveal tissue around the sclerotomy (Figure 2B). Notably, foreign forceps instead of intraocular micro-forceps should be used to grasp the slippery EPRL pieces tightly.

Considering the fact that EPRL is implanted mainly in high myopia, especially in eyes over −18D, serious concerns should be raised for zonular defect-associated complications. Comprehensive estimation for zonules should be performed as thoroughly as possible before surgery to rule out unsuitable patients. Very careful manipulation during operation is also advocated due to the potential weakness of the zonular of high myopic patients. Additionally, prolonging follow-up for monitoring the position of EPRL is also necessary for early detecting slight tilt of EPRL. In our case, EPRL fell into the vitreous cavity from posterior chamber two after the EPRL tilt was observed. Therefore, once tilt occurs, especially inferiorly, extraction of EPRL should be performed as soon as possible. Meanwhile, the patient should be suggested to reduce activity and keep a corresponding body position to avoid the aggravation of EPRL dislocation.

All in all, delayed dislocation of EPRL into the vitreous cavity due to zonular defects is a rare but severe complication of this procedure. Even though this complication can be successfully treated by pars plana vitrectomy with EPRL removal, concerns about zonules-related complications pre-operatively, intraoperatively, and post-operatively must be raised in the practice of EPRL implantation in the future.

## Data Availability Statement

The original contributions presented in the study are included in the article/Supplementary Material, further inquiries can be directed to the corresponding author/s.

## Ethics Statement

The studies involving human participants were reviewed and approved by Ethics Committee of the Second Affiliated Hospital of Zhejiang University. Written informed consent for participation was not required for this study in accordance with the national legislation and the institutional requirements.

## Author Contributions

JH, LZ, FZ, and XF: wrote and reviewed the manuscript. JH: collected materials. XF: designed the research and performed the surgery. All authors contributed to the article and approved the submitted version.

## Funding

This work was supported by grants from the Zhejiang Provincial Natural Science Foundation of China (No. LQ20H120010).

## Conflict of Interest

The authors declare that the research was conducted in the absence of any commercial or financial relationships that could be construed as a potential conflict of interest.

## Publisher's Note

All claims expressed in this article are solely those of the authors and do not necessarily represent those of their affiliated organizations, or those of the publisher, the editors and the reviewers. Any product that may be evaluated in this article, or claim that may be made by its manufacturer, is not guaranteed or endorsed by the publisher.
